# Lipid Peroxidation Regulators GPX4 and FSP1 as Prognostic Markers and Therapeutic Targets in Advanced Gastric Cancer

**DOI:** 10.3390/ijms25179203

**Published:** 2024-08-24

**Authors:** Kazuhiro Tamura, Yoshinobu Tomita, Takumi Kanazawa, Hajime Shinohara, Masayoshi Sakano, Sachiko Ishibashi, Masumi Ikeda, Mayumi Kinoshita, Junko Minami, Kurara Yamamoto, Yuki Kato, Asuka Furukawa, Shigeo Haruki, Masanori Tokunaga, Yusuke Kinugasa, Morito Kurata, Masanobu Kitagawa, Kenichi Ohashi, Kouhei Yamamoto

**Affiliations:** 1 Department of Human Pathology, Graduate School of Medical and Dental Sciences, Tokyo Medical and Dental University, Tokyo 113-8510, Japan; k.tamura.pth1@tmd.ac.jp (K.T.); 18cm127@s.bgu.jp (Y.T.); 23ms203@s.bgu.ac.jp (T.K.); mkinoshita@bgu.ac.jp (M.K.); jminami@toin.ac.jp (J.M.); ykatpth1@tmd.ac.jp (Y.K.); a.tajima.pth1@tmd.ac.jp (A.F.); kohashi.pth1@tmd.ac.jp (K.O.); 2 Department of Clinical Laboratory Medicine, Faculty of Health Science Technology, Bunkyo Gakuin University, Tokyo 113-8668, Japan; 3 Department of Gastrointestinal Surgery, Graduate School of Medical and Dental Sciences, Tokyo Medical and Dental University, Tokyo 113-8510, Japan; h.shino524@gmail.com (H.S.); masakun04222@gmail.com (M.S.); harukishigeo@me.com (S.H.); tokunaga.srg1@tmd.ac.jp (M.T.); kinugasa.srg1@tmd.ac.jp (Y.K.); 4 Department of Comprehensive Pathology, Graduate School of Medical and Dental Sciences, Tokyo Medical and Dental University, Tokyo 113-8510, Japan; sishpth2@tmd.ac.jp (S.I.); mikepth2@tmd.ac.jp (M.I.); kurata.pth2@tmd.ac.jp (M.K.); kitagawa@nakanosogo.or.jp (M.K.)

**Keywords:** gastric cancer, chemotherapy, lipid peroxidation, ferroptosis

## Abstract

Gastric cancer is one of the most common cancers worldwide, and new therapeutic strategies are urgently needed. Ferroptosis is an intracellular iron-dependent cell death induced by the accumulation of lipid peroxidation, a mechanism different from conventional apoptosis and necrosis. Therefore, induction of ferroptosis is expected to be a new therapeutic strategy. Glutathione peroxidase 4 (GPX4) and ferroptosis suppressor protein 1 (FSP1) have been identified as the major inhibitors of ferroptosis. Herein, we performed immunohistochemistry for GPX4, FSP1, and 4-HNE using tissues from patients with gastric cancer and investigated the relationship between these factors and prognosis. Patients with high GPX4 expression or high GPX4 expression and low 4-HNE accumulation tended to have a poor prognosis (*p* = 0.036, 0.023), whereas those with low FSP1 expression and high 4-HNE accumulation had a good prognosis (*p* = 0.033). The synergistic induction of cell death by inhibiting GPX4 and FSP1 in vitro was also observed, indicating that the cell death was non-apoptotic. Our results indicate that the expression and accumulation of lipid peroxidation-related factors play an important role in the clinicopathological significance of gastric cancer and that novel therapeutic strategies targeting GPX4 and FSP1 may be effective in treating patients with gastric cancer who have poor prognosis.

## 1. Introduction

Gastric cancer (GC) is one of the cancers with the most unfavorable prognosis. GC was the fifth most frequent (5.6%) and fourth deadliest (7.7%) cancer in 2020 [1]. Although a regimen combining platinum-based drugs and fluoropyrimidines is recommended as first-line chemotherapy for advanced GC, drug resistance remains a significant problem [2,3,4]. In recent years, the use of molecular-targeted agents and immune checkpoint inhibitors has been explored; however, their therapeutic efficacy remains limited owing to issues such as heterogeneity in target gene expression. Consequently, an urgent need exists to develop novel therapeutic strategies for treating advanced GC.

Cancer cells are resistant to programmed cell death, such as apoptosis, and can develop resistance to existing chemotherapies. Ferroptosis, discovered in 2012, is an intracellular, iron-dependent cell death induced by the accumulation of peroxidized lipids [5]. This mechanism of cell death is different from apoptosis, necrosis, and autophagy. It also effectively induces cell death in cancer cells that are resistant to apoptosis and is expected to be a new strategy for cancer treatment [6,7]. Consequently, research into the molecular mechanism of ferroptosis has accelerated in recent years with the identification of two key ferroptosis suppressor factors: glutathione peroxidase 4 (GPX4) and ferroptosis suppressor protein 1 (FSP1) [8,9,10].

GPX4 is regarded as a pivotal antioxidant enzyme in mammals, largely because of its distinctive capacity to directly reduce peroxidized phospholipids using reduced glutathione (GSH) as a cofactor [8]. FSP1 was previously known as apoptosis-inducing factor mitochondria-associated 2 (AIFM2). Recent studies have demonstrated that it inhibits ferroptosis. FSP1 inhibits the accumulation of lipid peroxides at the plasma membrane using coenzyme Q10 (CoQ10) as a cofactor; this pathway is characterized by the glutathione-independent inhibition of ferroptosis [9,10]. GC acquires resistance to ferroptosis and chemotherapy via GPX4 [11,12,13], and the overexpression of FSP1 may be involved in the poor prognosis of patients and immune cell infiltration in tumors [14]. Nevertheless, no reports exist on the use of immunostaining for 4-HNE, GPX4, and FSP1 in GC to evaluate prognosis. Furthermore, the molecular mechanism of cell death associated with GPX4 and FSP1 in GC has not yet been investigated in detail.

In this study, we investigated the relationship between lipid peroxidation regulators and GC prognosis, their association with cell death, and the potential for new therapeutic strategies.

## 2. Results

### 2.1. Immunohistochemistry

**Figure 1:**
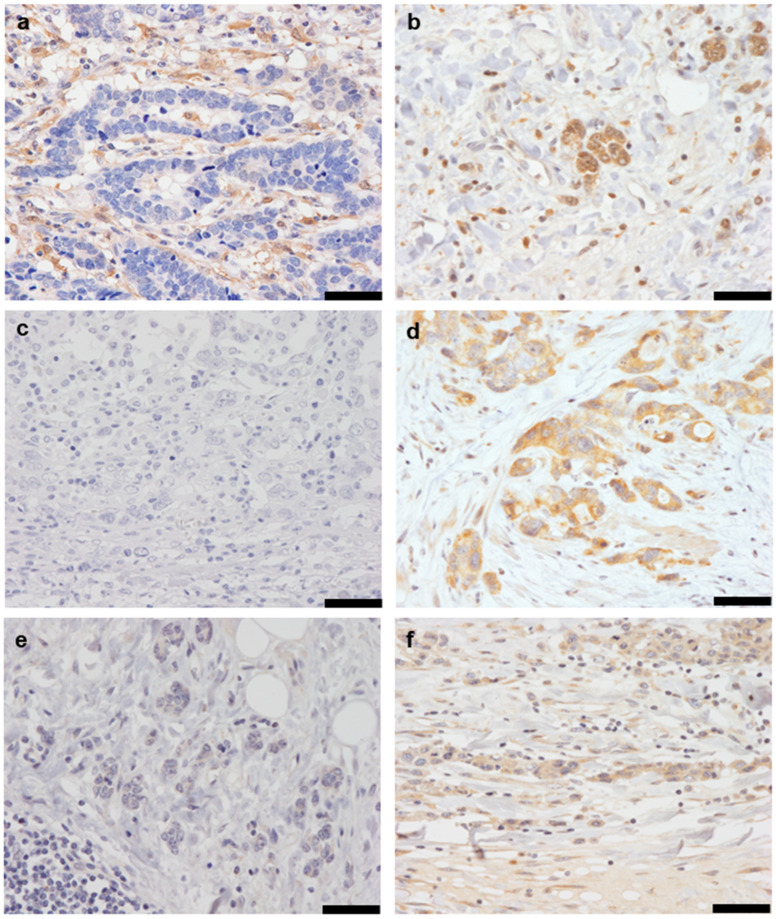
Immunostaining of gastric cancer tissue for GPX4 (**a**,**b**), FSP1 (**c**,**d**), and 4-HNE (**e**,**f**). (**a**,**b**) Representative weak (**a**) and strong (**b**) staining in GPX4 immunostaining. (**c**,**d**) Representative weak (**c**) and strong (**d**) staining in FSP1 immunostaining. (**e**,**f**) Representative weak (**e**) and strong (**f**) staining in 4-HNE immunostaining. The magnification is 200×, and the scale bar is 50 μm.

Immunohistochemistry was performed to analyze the accumulation of GPX4, FSP1, and 4-HNE in GC tissues. Staining intensity was evaluated and classified as either low or high. Representative images of staining are shown (Figure 1). Among the 163 patients, 106 (65.0%) showed low expression and 57 (35.0%) showed high expression of GPX4, whereas 101 (62.0%) showed low expression and 62 (38.0%) showed high expression of FSP1. Accumulation of 4-HNE was low in 107 patients (65.6%) and high in 56 patients (34.4%).

### 2.2. Relationship between Lipid Peroxidation Regulators and 4-HNE

The associations between GPX4 expression and 4-HNE accumulation and that between FSP1 expression and 4-HNE accumulation were analyzed (Table 1). More cases of low 4-HNE accumulation were observed in the group with low GPX4 expression compared to the group with high GPX4 expression (77/106, 72.6% vs. 30/57, 52.6%). Although we found a significant association between GPX4 expression and 4-HNE accumulation (*p* = 0.015), the association was weak (V = 0.201). In addition, more cases of low 4-HNE accumulation were observed in the group with low FSP1 expression than in the group with high FSP1 expression (76/101, 75.2% vs. 31/62, 50.0%). Although we found a significant association between FSP1 expression and 4-HNE accumulation (*p* = 0.001), the association was weak (V = 0.258).

### 2.3. Association of Lipid Peroxidation-Related Factors with Clinicopathological Factors

The relationships among GPX4 expression, FSP1 expression, and 4-HNE accumulation in GC tissues and various clinicopathological factors were investigated (Table 2). A significant correlation was observed between differentiation and 4-HNE accumulation (*p* = 0.017), with poorly differentiated adenocarcinomas demonstrating a higher percentage of low 4-HNE accumulation. A significant association was also observed between the mode of invasion and GPX4 and FSP1 expression (*p* = 0.031 and *p* = 0.021, respectively), and cases with infiltrative invasion were more likely to have high GPX4 and FSP1 expression. In addition, FSP1 expression was significantly associated with lymphatic invasion (*p* = 0.0496), with a higher proportion of cases showing higher FSP1 expression in the lymphatic invasion-positive group.

### 2.4. Survival Analysis

**Figure 2:**
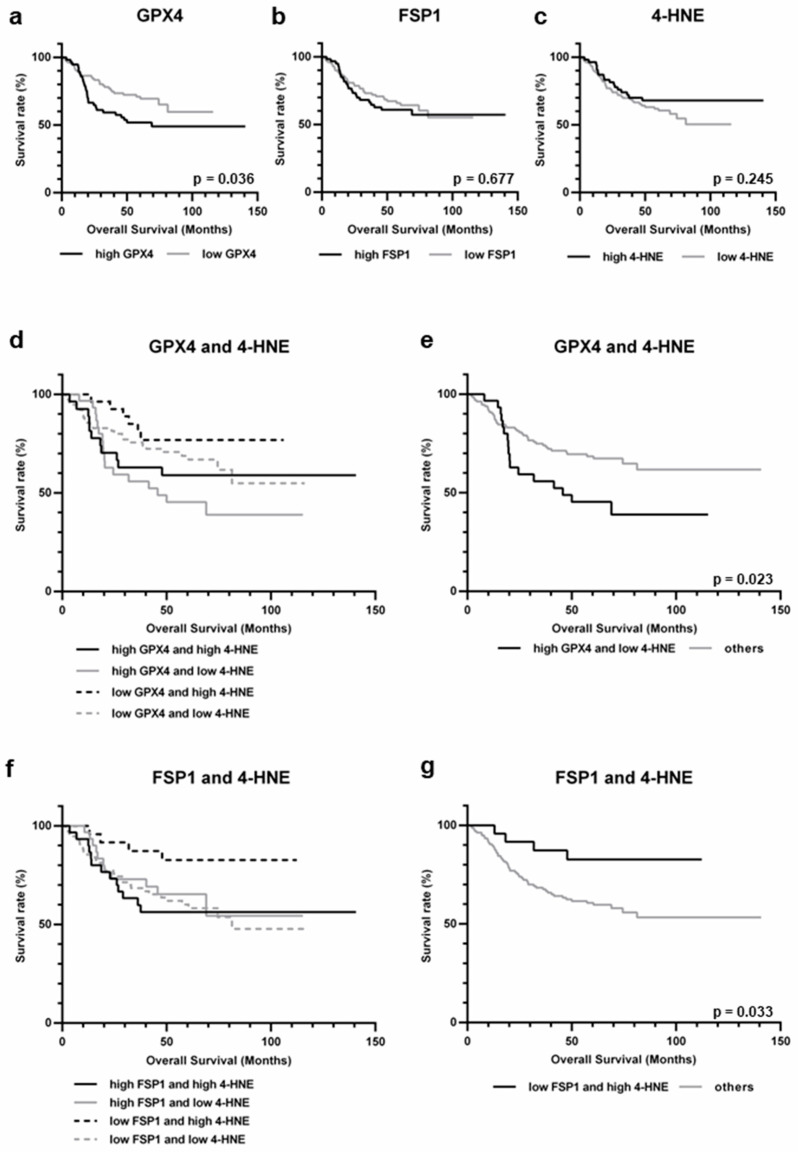
Kaplan–Meier analysis of gastric cancer, stratified by result of immunohistochemistry. (**a**) High expression of GPX4 correlated with poor prognosis (*p* = 0.036). (**b**) No significant difference was observed between FSP1 high and low groups (*p* = 0.677). (**c**) No significant difference was observed between 4-HNE high and low groups (*p* = 0.245). (**d**) Stratification based on GPX4 and 4-HNE. (**e**) The prognosis of high GPX4 expression and low 4-HNE accumulation was worse than that of the others (*p* = 0.023). (**f**) Stratification based on FSP1 and 4-HNE. (**g**) The prognosis of low FSP1 expression and high 4-HNE accumulation was better than that of the others (*p* = 0.033).

OS was analyzed based on stratified GPX4 and FSP1 expression and 4-HNE accumulation. The group with high GPX4 expression had a significantly poorer prognosis (*p* = 0.036) (Figure 2a). No significant association was observed between FSP1 expression, 4-HNE accumulation, and prognosis (Figure 2b,c). Next, OS was analyzed by combining GPX4 expression with 4-HNE accumulation and FSP1 expression with 4-HNE accumulation. Patients with high GPX4 expression and low 4-HNE accumulation had a poorer prognosis (*p* = 0.023) (Figure 2d,e). Conversely, patients with low FSP1 expression and high 4-HNE accumulation had a better prognosis (*p* = 0.033) (Figure 2f,g). Univariate analysis of clinicopathological factors showed that lymph node metastasis, lymphatic invasion, and venous invasion were significantly associated with prognosis (Table 3). Multivariate analysis of these factors, including GPX4 expression, FSP1 expression, and 4-HNE accumulation, revealed that low FSP1 expression and high 4-HNE accumulation were independent favorable prognostic factors (Table 4).

### 2.5. Effect of GPX4 on Cell Proliferation in GC Cell Lines

**Figure 3:**
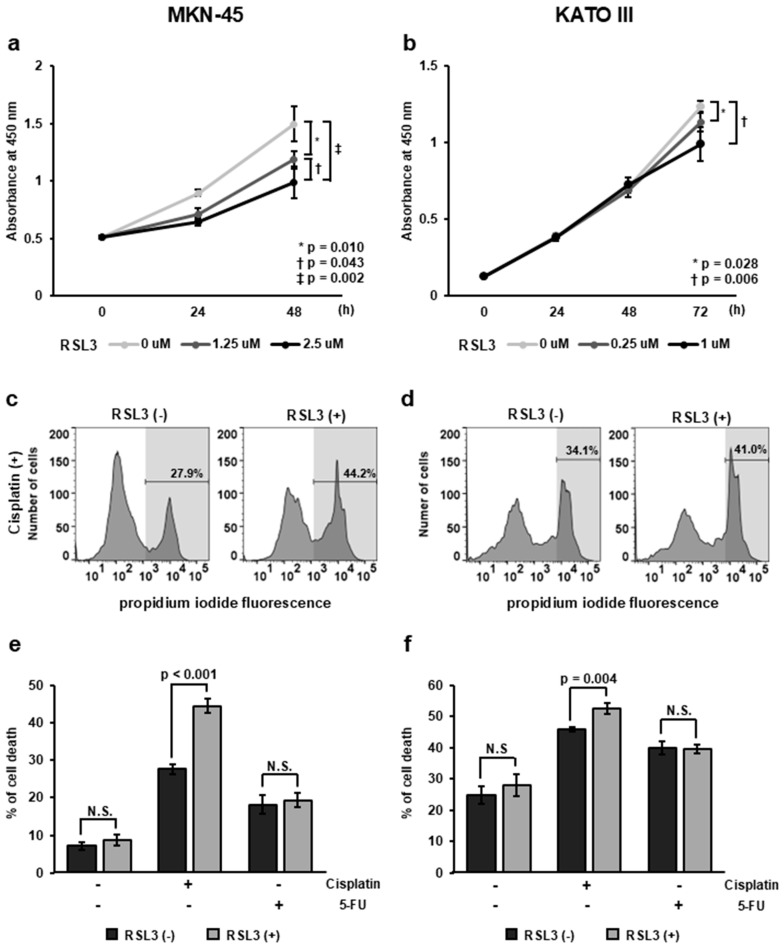
Effect of GPX4 on the proliferation and sensitivity to apoptosis-inducing drugs. (**a**,**b**) Cell proliferation assay under treatment with RSL3, a GPX4 inhibitor. Cell proliferation was inhibited in both MKN-45 (**a**) and KATO III (**b**) cells (MKN-45, *p* = 0.010 at 1.25 μM RSL3, *p* = 0.002 at 2.5 μM RSL3, 48 h after treatment; KATO III, *p* = 0.028 at 0.25 μM RSL3, *p* = 0.006 at 1 μM RSL3, 72 h after treatment). (**c**,**d**) Histogram of flow cytometry performed with propidium iodide 48 h after administration of RSL3, a GPX4 inhibitor, and cisplatin, an apoptosis inducer. (**e**,**f**) Cell death rate measured by flow cytometry. In both MKN-45 and KATO III cells, RSL3 treatment significantly increased cell death with cisplatin treatment (MKN-45, *p* = 0.006; KATO III, *p* = 0.043).

Previous studies suggested that high GPX4 expression is associated with poor prognosis. To investigate whether GPX4 contributes to the proliferation of GC cells, we examined whether RSL3, an inhibitor of GPX4, suppressed cell proliferation in GC cell lines. The results showed that, at 48 h, both 1.25 μM and 2.5 μM concentrations of RSL3 significantly inhibited cell proliferation in MKN-45 cells compared to the untreated group (*p* = 0.010, *p* = 0.002; Figure 3a). The inhibition of cell proliferation increased as the concentration increased. In KATO III cells, significant inhibition of cell proliferation was observed at 72 h for both the 0.25 μM and 1 μM of RSL3 concentrations compared to the untreated group (*p* = 0.028, *p* = 0.006; Figure 3b).

### 2.6. Effect of GPX4 on Apoptosis-Inducing Cell Death

To determine how GPX4 contributes to the survival of GC cells, we examined whether RSL3 treatment altered the apoptosis-inducing cell death rate caused by cisplatin or 5-FU. RSL3 significantly enhanced cisplatin-induced cell death in both MKN-45 (Figure 3e) and KATOIII cells (Figure 3f) (MKN-45, *p* = 0.006; KATO III, *p* = 0.043). Cell death induced by 5-FU was not enhanced by RSL3.

### 2.7. Synergistic Effects of GPX4 and FSP1 Inhibition on Cell Death Induction

**Figure 4:**
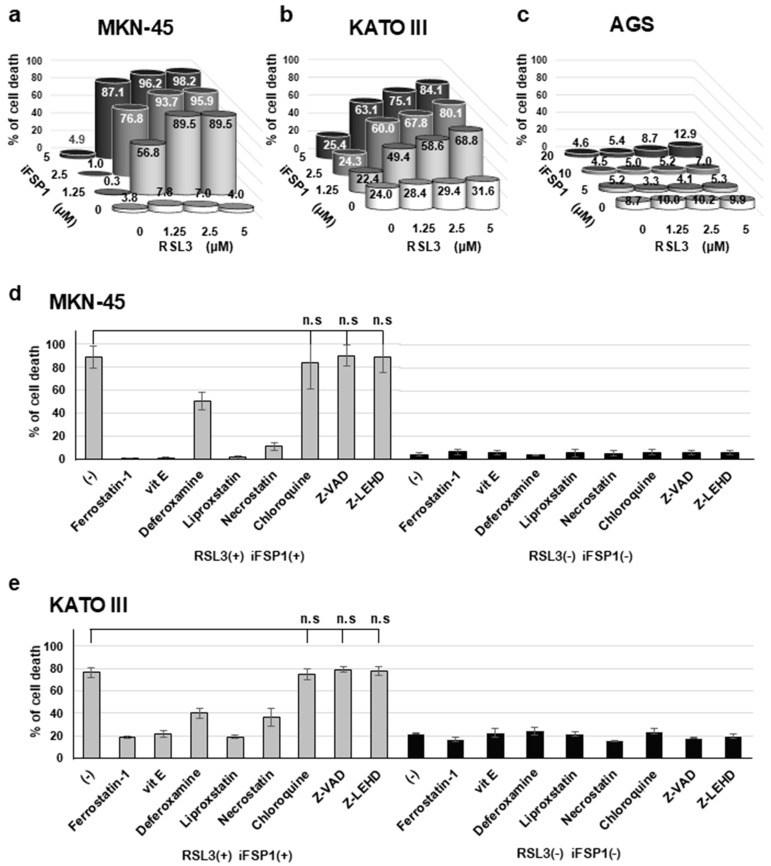
Synergistic effects of GPX4 and FSP1 inhibition on cell death induction. (**a**–**c**) Cell death rates with administration of RSL3, an inhibitor of GPX4, and iFSP1, an inhibitor of FSP1. In MKN-45 (**a**) and KATO III (**b**) cells, the synergistic effects on cell death induction were observed; in AGS (**c**) cells, combined treatment did not dramatically increase the cell death rate. (**d**,**e**) Investigation of type of cell death by cell death inhibitors. Cell death was fully inhibited by ferrostatin-1, vitamin E (vit E), liproxstatin, ferroptosis inhibitors, and necrostatin, necroptosis inhibitor. Cell death was partially inhibited by deferoxamine, an iron chelator.

To examine the contributions of GPX4 and FSP1, both lipid peroxidation regulators, to cell survival, we administered RSL3, an inhibitor of GPX4, and iFSP1, an inhibitor of FSP1, and assessed the cell death rates. In MKN-45 cells, single-agent induced cell death by only approximately 4.9%, even at high concentrations, whereas simultaneous administration of these agents induced cell death dramatically, up to 98.2% (Figure 4a). In KATO III cells, the cell death rate was not different from the baseline rate of approximately 31.6% with single-agent administration, whereas combined treatment induced a dramatic cell death rate of up to 84.1% (Figure 4b). Conversely, no synergistic effects were observed in AGS cells (Figure 4c).

### 2.8. Suppression of GPX4 and FSP1 Co-Inhibition-Induced Cell Death by Ferroptosis Inhibitors

To evaluate the type of cell death induced by the combined administration of RSL3 and iFSP1, we examined whether cell death was inhibited by the administration of cell death inhibitors (Figure 4d,e). Cell death was inhibited to a rate comparable to that in the RSL3 and iFSP1 non-treated groups by ferrostatin-1 and liproxstatin, both ferroptosis inhibitors; vitamin E, the antioxidant factor; and necrostatin, a necrosis inhibitor. Interestingly, the iron chelator deferoxamine only partially inhibited cell death.

## 3. Discussion

GC has a poor prognosis. The effectiveness of treatment for advanced GC is limited due to resistance to chemotherapy. In addition to common chemotherapies such as 5-FU and cisplatin, molecular-targeted drugs and immune checkpoint inhibitors have been used for treatment, but their effectiveness is limited. Therefore, an urgent need exists to develop novel therapeutic strategies.

This study identified several associations between clinicopathological factors and the expression and accumulation of lipid peroxidation-related factors. The high GPX4 expression group and high GPX4 expression with low 4-HNE accumulation group tended to have a poor prognosis, whereas the low FSP1 expression with high 4-HNE accumulation group tended to have a good prognosis. In GC, previous reports have shown an association between high expression of GPX4 [12,13] and FSP1 and poor prognosis [14]. In this study, we combined the expression of GPX4 and FSP1 with the accumulation of 4-HNE to clarify their relationship with prognosis. If 4-HNE reflects the accumulation of lipid peroxide, based on the positive effect of GPX4 expression on cell proliferation and the negative effect on apoptosis induction, the inhibitory effect of lipid peroxidation by GPX4 and FSP1 may positively affect cancer cell proliferation and survival, leading to poor prognosis by promoting disease progression and acquiring resistance to chemotherapy. In the GPX4-positive group, which had a significantly poorer prognosis, the prognosis may be improved by enhancing the postoperative chemotherapy regimen. In addition, since GPX4 was suggested to be involved in the resistance to cisplatin, a more effective therapeutic effect may be possible by using a first-line chemotherapy regimen other than cisplatin.

Regarding pathological factors, cancers had a higher invasive proliferative potential in the group with higher expression of lipid peroxidation regulators. In recent years, the association between epithelial-mesenchymal transition (EMT) and the invasive proliferative potential of cancer has attracted much attention. High expression of ZEB1, an EMT regulator, increases dependence on GPX4 [15], suggesting that the association between lipid peroxidation regulators and EMT may contribute to the invasive growth potential of GC. In addition, matrix metalloproteinases (MMP) are among the factors required for the invasive growth of cancer [16,17]. Although no reports show a direct relationship between lipid peroxidation-related factors and MMPs, oxidative stress increases MMP-1 expression [18], suggesting a possible association between lipid peroxidation-related factors and MMP expression or invasive growth potential.

Immunostaining showed a trend toward higher expression of lipid peroxidation regulators in the group with higher 4-HNE accumulation. If the 4-HNE immunostaining results reflect the accumulation of lipid peroxidation, it is assumed that the high expression of lipid peroxidation regulators reduces the accumulation of 4-HNE. Kinowaki et al. reported that immunostaining for DLBCL showed a trend toward lower accumulation of 8-OHdG, a marker of oxidative stress, in the group with high GPX4 expression [19]. Conversely, Gao et al. reported that Nrf2 signaling induces glutathione and GPX4 under oxidative stress in NAFLD [20]. Regarding the quantitative relationship between 4-HNE and lipid peroxidation regulators, FSP1 expression is reportedly induced by increased intracellular concentration of 4-HNE in a DLBCL cell line [21]. These reports suggest that oxidative stress and 4-HNE accumulation inversely regulate the expression of lipid peroxidation regulators, and a similar mechanism may be involved in GC. Watabe et al. reported that 4-HNE accumulation in hepatocellular carcinoma is regulated by the differential metabolic activity of 4-HNE through SMARCA4, regardless of the expression of GPX4, FSP1, or GCH1 [22]. The immunostaining results for 4-HNE may have been influenced by its metabolic pathway and not only by the level of lipid peroxidation. Further, miR-522, secreted by cancer-associated fibroblasts (CAF), reduces the accumulation of lipid peroxide in GC [23], suggesting that both the expression of lipid peroxidation regulators in tumor cells and tumor microenvironment regulates 4-HNE accumulation in cancer cells.

A synergistic effect on cell death induction was observed by co-inhibition of GPX4 and FSP1 in a GC cell line. In experiments with cell death inhibitors, the apoptosis inhibitors Z-VAD and Z-LEHD and autophagy inhibitor chloroquine did not inhibit this cell death. However, the necroptosis inhibitor necrostatin inhibited cell death, similar to ferroptosis inhibitors. This suggests that the cell death may be related to the necroptosis pathway. Nevertheless, necrostatin suppresses ferroptosis by exerting an antioxidant effect independent of key factors of necroptosis, such as RIPK1 and IDO [24], suggesting that this cell death may be similar to ferroptosis in one way. In squamous cell carcinoma of the lung cell lines, synergistic cell death resulting from GPX4 and FSP1 inhibition by RSL3 and iFSP1 is through iron-dependent ferroptosis [25]. Since many reports have shown that iron chelators fully inhibit cell death [5,12,26], the cell death observed in this study may be ferroptosis-like, fully inhibited by ferroptosis inhibitors, but partially lacks iron dependence. The Fenton reaction, the main trigger of lipid peroxidation, occurs in the presence of metal ions with multiple redox states, such as copper, cobalt, and manganese, in addition to iron [27]. Furthermore, peroxynitrite, a potent oxidant in vivo, causes iron-independent peroxidation of membrane lipids [28]. These non-iron ions may induce ferroptosis-like cell death by mediating Fenton and similar oxidative reactions.

There are challenges associated with the clinical application of ferroptosis, as many elements of the mechanism of ferroptosis remain unclear. In this study, some cancer cell lines were unable to induce ferroptosis sufficiently by inhibiting GPX4 and FSP1. In this context, cuproptosis, a recently discovered copper-dependent cell death pathway, is attracting attention. The intracellular accumulation of copper ions disrupts proteins related to the tricarboxylic acid (TCA) cycle and electron transport chain, leading to cell death by suppressing mitochondrial respiration [29]. Glutathione is a potential therapeutic target for the treatment of cuproptosis. Glutathione is a factor that regulates and inhibits cuproptosis by binding to copper ions [29]. It also inhibits ferroptosis as an antioxidant factor [8] and induces cisplatin resistance by binding to platinum [30]. These results suggest that enhanced induction of cell death by GPX4 inhibition was observed only with cisplatin and not with 5-FU. Co-induction of ferroptosis and cuproptosis by targeting the glutathione-GPX4 system may be a more effective strategy to induce cell death in cancers that are currently resistant to various types of cell death.

This study had some limitations. First, this was a retrospective study using GC specimens resected at a single institution with a limited number of cases. This may affect the generalizability of the results. A multicenter prospective cohort study is expected to increase generalizability. Since other lipid peroxidation-related factors, such as GCH1—which is reportedly associated with ferroptosis, were not examined in this study, we cannot rule out the possibility that these factors may influence clinicopathological factors. It is desirable to search for associations between these factors and clinicopathological factors. We were unable to evaluate enzyme substrates such as glutathione and CoQ10 and cannot rule out the possibility that the concentration and metabolism of these substrates may be related to cell growth and survival. It is desirable to evaluate these substrates as well, including their relationship to lipid peroxidation regulators. In this study, the functions of lipid peroxidation regulators were examined by administering inhibitors to cell lines; however, the inhibitors may affect enzymes other than the targeted enzymes non-specifically. To achieve more specific enzyme inhibition, molecular biology techniques using siRNA or the CRISPR-Cas9 system are desirable.

## 4. Materials and Methods

### 4.1. Patients and Samples

Formalin-fixed paraffin-embedded (FFPE) tissues were obtained from 163 patients with pT3 or pT4 GC who underwent surgical resection at the Tokyo Medical and Dental University Hospital between 2003 and 2016 without preoperative chemotherapy. This study was approved by our Ethics Committee (approval number: M2000-1706).

### 4.2. Immunohistochemistry

The FFPE tissue, including the deepest part of the tumor, was sliced to a thickness of 3 μm and affixed to silane-coated glass (Matsunami Glass Industries, Osaka, Japan). The tissues were then deparaffinized and rinsed. For antigen retrieval, specimens for GPX4 and 4-HNE staining were treated with pH 6.0 citrate buffer for 20 min at 97 °C, and specimens for FSP1 staining were treated with pH 9.0 HISTOFINE (Nichirei Biosciences, Tokyo, Japan) for 40 min at 97 °C in a microwave oven. Endogenous peroxidase was inactivated by the addition of a hydrogen peroxide solution (3%) in methanol. Blocking of nonspecific reactions was performed using 2.5% Normal Horse Serum (Vector Laboratories, Newark, CA, USA). Primary antibodies were added at 4 °C for 24 h. GPX4 was sensitized by the iAEP method (Leica Biosystems, Nussloch, Germany), while FSP1 and 4-HNE were sensitized by the ABC method (Vector Laboratories). Color development was performed using diaminobenzidine (Nichirei Biosciences). The nuclei were stained with Mayer’s hematoxylin (Muto Pure Chemicals, Tokyo, Japan), and the specimens were dehydrated, permeabilized, and sealed. Details of the antibodies are presented in the Appendix A (Table A1). We compared the intensity of positivity to that of macrophages in the specimen. We defined a case with a stronger positive intensity than macrophages as having a high expression and a case with an equal or weaker positive intensity as having a low expression.

### 4.3. Clinicopathologic Analysis

Eight clinicopathological factors were analyzed. The factors were age (<60 years vs. ≥60 years), gender (male vs. female), differentiation (highly differentiated vs. poorly differentiated), mode of invasion (expansive vs. infiltrative), lymph node metastasis (negative vs. positive), lymphatic invasion (negative vs. positive), venous invasion (negative vs. positive), and depth of invasion (pT3 vs. pT4). Overall survival (OS) was compared for the following clinicopathological factors: GPX4 expression, FSP1 expression, and 4-HNE accumulation.

### 4.4. Cell Lines

KATO III, MKN-45, and AGS cells derived from human GC were used for in vitro analysis. These cells were obtained from the Japanese Collection of Research Bioresources. KATO III cells were cultured in IMDM (containing L-glutamine, phenol red, HEPES, sodium pyruvate) medium (FUJIFILM Wako Pure Chemical Corporation, Osaka, Japan) with 20% fetal bovine serum (FBS). MKN-45 cells were cultured in RPMI-1640 (containing L-glutamine, phenol red) medium (FUJIFILM Wako Pure Chemical Corporation) with 10% FBS. AGS cells were cultured in a DMEM high-glucose medium containing L-glutamine, phenol red (Wako Pure Chemical Industries), and 10% FBS. These mediums were added with 1% penicillin-streptomycin. The cells were passaged every 3–4 days at a ratio of 1:10.

### 4.5. Analysis of the Effect of GPX4 Inhibitors on Cell Proliferation

The effect of RSL3 (MedChemExpress, Tokyo, Japan), a GPX4 inhibitor, on the proliferation of MKN-45 and KATO III cells was evaluated. Each cell line was seeded into 96-well culture plates at a density of 1 × 10^3^ cells per well and subsequently cultured at 37 °C for 24 h. MKN-45 cells were exposed to RSL3 at the following concentrations: 0, 1.25, and 2.5 µM, and cell proliferation was assessed at 0, 24, and 48 h after RSL3 addition using the Cell Counting Kit-8 (DOJINDO LABORATORIES, Kumamoto, Japan). KATO III cells were cultured at 37 °C for 24 h and subsequently exposed to RSL3 at the following concentrations: 0, 0.25, and 1 µM. Subsequently, cell proliferation was assessed at 0, 24, 48, and 72 h after RSL3 addition. Following the addition of the Cell Counting Kit-8 reagent, the cells were incubated at 37 °C for an additional 1.5 h. The absorbance was measured at 450 nm using a microplate reader (ELx808; Agilent Technologies, Inc., Santa Clara, CA, USA).

### 4.6. Analysis of the Effect of GPX4 Inhibitors on Apoptosis-Inducing Cell Death

Cell death was evaluated in MKN-45 and KATO III cells using RSL3, Cisplatin (FUJIFILM Wako Pure Chemical Corporation), or 5-FU (FUJIFILM Wako Pure Chemical Corporation). Each cells were seeded in 24-well culture plates at a density of 1 × 10^5^ cells/well and cultured at 37 °C for 24 h. MKN-45 cells were exposed to RSL3: 0 µM and 2.5 µM and cisplatin: 0 µM and 100 µM, or RSL3: 0 µM and 2.5 µM and 5-FU: 0 µg/mL and 400 µg/mL. KATO III cells were exposed to RSL3: 0 µM and 1 µM and cisplatin: 0 µM and 100 µM, or RSL3: 0 µM and 1 µM and 5-FU: 0 µg/mL and 400 µg/mL. At 48 h after exposure, cells were collected and labeled with propidium iodide (Sigma-Aldrich Japan, Tokyo, Japan), and the percentage of dead cells was calculated using a BD FACSCanto™ flow cytometer (Becton Dickinson and Company, Franklin Lakes, NJ, USA).

### 4.7. Analysis of the Effects of GPX4 Inhibitor and FSP1 Inhibitor on Cell Death

Cell death was evaluated in MKN-45, KATO III, and AGS cells treated with RSL3 and iFSP1 (an FSP1 inhibitor). After 24 h of incubation at 37 °C, the cells were treated with 0, 1.25, 2.5, and 5 µM of RSL3 and 0, 1.25, 2.5, and 5 µM of iFSP1. The cell death rates were measured after 48 h of incubation. For MKN-45 and AGS cells, the cell death rate was measured using the Cytotoxicity LDH Assay Kit-WST (DOJINDO LABORATORIES). In 96-well plates (CORNING, Corning, NY, USA), 3 × 10^4^ cells were seeded. After 48 h of exposure, 100 μL of working solution was added, and after shading from light for 30 min at room temperature, 50 μL of stop solution was added to stop the reaction. The absorbance was measured at 490 nm using a microplate reader ELx808 (BioTek, Winooski, VT, USA). The death rate of KATO III cells was measured according to the aforementioned method, “Analysis of the effect of GPX4 inhibitors on apoptosis-inducing cell death”.

### 4.8. Analysis of GPX4 and FSP1 Inhibitor-Induced Cell Death

The effects of these inhibitors on cell death induced by RSL3 and iFSP1 were examined in MKN-45 and KATO III cells. MKN-45 cells were treated with 2.5 µM of RSL3, 20 µM of iFSP1, and the following cell death inhibitors: 25 µM of Z-VAD-FMK (Peptide Institute, Osaka, Japan), 20 µM of Z-LEHD-FMK (Medical Biological Laboratory, Nagoya, Japan), 100 µM of necrostatin (AdipoGen Life Science, San Diego, CA, USA), 1 µM of chloroquine (Sigma-Aldrich, St Louis, MO, USA), 20 µM of ferrostatin-1 (Sigma-Aldrich), 250 µM of liproxstatin (Sigma-Aldrich), 200 µM of deferoxamine (Sigma-Aldrich), and 100 µM of vitamin E (FUJIFILM Wako). KATOIII cells were treated with 5 µM of RSL3, 5 µM of iFSP1, and the same cell death inhibitors as MKN-45 cells. However, the concentrations were partially different: deferoxamine was administered at 20 µM and vitamin E at 500 µM. The method of measurement was the same as previously described in “Analysis of the effect of GPX4 inhibitors on apoptosis-inducing cell death”.

### 4.9. Statistical Analysis

For the analysis of the association between lipid peroxidation regulators and 4-HNE immunological results and between lipid peroxidation-related factors and clinicopathological factors, we selected Fisher’s exact test for the association between the two groups without correspondence. The log-rank test was used for survival analysis. These analyses were performed using GraphPad PRISM ver. 6 (GraphPad Software, Inc., Boston, MA, USA). For univariate and multivariate analyses, the Cox proportional hazard model was selected because of the inclusion of a temporal component. These analyses were performed using EZR software version 1.68 (Saitama Medical Center, Jichi Medical University, Saitama, Japan). Statistical significance was set at *p* < 0.05. 

Based on the strength of the association, Cramer’s coefficient of association was calculated using Excel 2019 (Microsoft Corporation, Redmond, WA, USA).

## 5. Conclusions

The clinicopathological and molecular significance of the expression and accumulation of lipid peroxidation-related factors in GC has been partially elucidated. GPX4 and FSP1 were identified as prognostic markers, and these markers may assist in the choice of treatment strategy and contribute to the improvement in the life expectancy of patients. The induction of ferroptosis-like cell death by the inhibition of GPX4 and FSP1 may be an effective treatment for GC refractory to conventional chemotherapy, and a combination of unknown cell death mechanisms may improve the prognosis of patients with GC. Further research on lipid peroxidation-associated cell death and its clinical applications, including detailed mechanistic and in vivo studies, is highly anticipated.

## Figures and Tables

**Table 1:** Association between accumulation of 4-HNE and expression of lipid peroxidation regulators.

		GPX4			
		Low	High	V	*p*-Value
4-HNE	Low	77	30	0.201	**0.015**
	High	29	27		
		**FSP1**			
		**Low**	**High**	**V**	***p*** **-Value**
4-HNE	Low	76	31	0.258	**0.001**
	High	25	31		

GPX4: Glutathione peroxidase 4, FSP1: Ferroptosis suppressor protein 1, 4-HNE: 4-hydoxynonenal, V: Cramer’s coefficient of association.

**Table 2:** Association between clinicopathological characteristics and expression of GPX4, FSP1, and 4-HNE.

Characteristics	n	GPX4			FSP1			4-HNE		
		Low	High	*p*-Value	Low	High	*p*-Value	Low	High	*p*-Value
**Age**										
<60	32	24	8	0.219	24	8	0.106	21	11	1.000
≥60	131	82	49		77	54		86	45	
**Sex**										
Male	117	79	38	0.362	73	44	0.860	78	39	0.715
Female	46	27	19		28	18		29	17	
**Differentiation**										
well	63	36	27	0.129	40	23	0.869	34	29	0.017 *
poor	100	70	30		61	39		73	27	
**Pattern of invasion**										
expansive	96	69	27	0.031 *	67	29	0.021 *	63	33	1.000
infiltrative	67	37	30		34	33		44	23	
**Lymph node metastasis**										
negative	67	48	19	0.182	45	22	0.325	47	20	0.402
positive	96	58	38		56	40		60	36	
**Lymphatic invasion**										
negative	47	33	14	0.469	35	12	0.0496 *	35	12	0.148
positive	116	73	43		66	50		72	44	
**Vascular invasion**										
negative	21	16	5	0.330	17	4	0.059	16	5	0.332
positive	142	90	52		84	58		91	51	
**Depth of invasion**										
pT3	95	66	29	0.184	64	31	0.104	61	34	0.739
pT4 a	68	40	28		37	31		46	22	

GPX4: Glutathione peroxidase 4, FSP1: Ferroptosis suppressor protein 1, 4-HNE: 4-hydoxynonenal. *, *p* < 0.05.

**Table 3:** Univariate analysis of clinicopathological characteristics and expression of GPX4, FSP1, and 4-HNE.

Characteristics	n	HR	95% CI	*p*-Value
**Age**				
<60	32	0.69	0.34–1.40	0.304
≥60	131			
**Sex**				
Male	117	1.35	0.74–2.47	0.322
Female	46			
**Differentiation**				
well	63	0.70	0.41–1.21	0.200
poor	100			
**Mode of invasion**				
expansive	96	1.43	0.86–2.39	0.170
infiltrative	67			
**Lymph node metastasis**				
negative	67	2.63	1.44–4.81	0.002 *
positive	96			
**Lymphatic invasion**				
negative	47	2.52	1.28–4.98	0.008 *
positive	116			
**Vascular invasion**				
negative	21	10.07	1.39–72.68	0.022 *
positive	142			
**Depth of invasion**				
pT3	95	1.43	0.99–2.05	0.053
pT4a	68			
**GPX4**				
low	106	1.72	1.03–2.87	0.038 *
high	57			
**FSP1**				
low	101	1.12	0.66–1.88	0.677
high	62			
**4-HNE**				
low	107	0.72	0.41–1.26	0.247
high	56			
**GPX4 and 4-HNE**				
high GPX4 and low 4-HNE	30	0.52	0.30–0.92	0.025 *
others	133			
**FSP1 and 4-HNE**				
low FSP1 and high 4-HNE	25	2.87	1.04–7.93	0.042 *
others	138			

GPX4: Glutathione peroxidase 4, FSP1: Ferroptosis suppressor protein 1, 4-HNE: 4-hydoxynonenal. *, *p* < 0.05.

**Table 4:** Multivariate analysis of the clinicopathological characteristics and expression of GPX4, FSP1, and 4-HNE.

Characteristics	n	GPX4 Multivariate Analysis			GPX4 and 4-HNE Multivariate Analysis			FSP1 and 4-HNE Multivariate Analysis		
		HR	95% CI	*p*-Value	HR	95% CI	*p*-Value	HR	95% CI	*p*-Value
**Lymph node metastasis**										
negative	67	2.02	1.09–3.77	0.027 *	1.88	0.99–3.54	0.053	2.24	1.19–4.19	0.012 *
positive	96									
**Lymphatic invasion**										
negative	47	1.45	0.71–2.97	0.312	1.53	0.74–3.15	0.248	1.37	0.67–2.81	0.389
positive	116									
**Vascular invasion**										
negative	21	6.33	0.84–47.92	0.074	6.45	0.85–48.69	0.071	6.31	0.84–47.29	0.073
positive	142									
**GPX4**										
low	106	1.58	0.94–2.64	0.083	-	-	-	-	-	-
high	57									
**GPX4 and 4-HNE**										
high GPX4 and low 4-HNE	30	-	-	-	0.58	0.33–1.04	0.066	-	-	-
others	133									
**FSP1 and 4-HNE**										
low FSP1 and high 4-HNE	25	-	-	-	-	-	-	3.04	1.10–8.42	0.032 *
others	138									

GPX4: Glutathione peroxidase 4, FSP1: Ferroptosis suppressor protein 1, 4-HNE: 4-hydoxynonenal. *, *p* < 0.05.

## Data Availability

The original contributions presented in the study are included in the article, further inquiries can be directed to the corresponding authors.

## Appendix A

**Table A1:** Details of the antibodies.

Antibody	Host	Type	Clone	Source	Dilution
GPX4	Rabbit	monoclonal	EPNCIR144	Abcam	1:200
FSP1	Rabbit	polyclonal	-	Sigma	1:200
4-HNE	Mouse	monoclonal	HNEJ-2	JaICA	1:200

GPX4: Glutathione peroxidase 4, FSP1: Ferroptosis suppressor protein 1, 4-HNE: 4-hydoxynonenal, JaICA: Japan Institute for the Control of Aging, NIKKEN SEIL. Co., Ltd., Shizuoka, Japan.
